# The combination of preoperative celiac axis stenting and neoadjuvant chemotherapy in an elderly patient with pancreatic cancer: a case report

**DOI:** 10.1186/s40792-024-01857-2

**Published:** 2024-03-12

**Authors:** Susumu Doita, Hideki Aoki, Hiroki Kajioka, Kohji Tanakaya, Kenji Kawamoto

**Affiliations:** 1 Department of Surgery, National Hospital Organization Iwakuni Clinical Center, 1-1-1 Atago-Machi, Iwakuni-City, Yamaguchi 740-8510 Japan; 2 Department of Cardiovascular Medicine, National Hospital Organization Iwakuni Clinical Center, 1-1-1 Atago-Machi, Iwakuni-City, Yamaguchi 740-8510 Japan

**Keywords:** Celiac artery stenosis, Pancreaticoduodenectomy, Preoperative stenting, Neoadjuvant chemotherapy

## Abstract

**Background:**

Celiac axis stenosis (CAS) is frequently observed in patients undergoing pancreaticoduodenectomy (PD). This poses challenges because of the potential disruption of the hepatic arterial blood flow.

**Case presentation:**

We present the case of an 81-year-old woman diagnosed with pancreatic head cancer and severe CAS caused by calcification. The patient received neoadjuvant chemotherapy (NAC) and underwent preoperative endovascular stenting of the celiac axis to restore blood flow. After two cycles of NAC, subtotal stomach-preserving PD was performed. An intraoperative assessment of the hepatic arterial blood flow determined that it was well maintained. PD was performed using the standard technique; specialized techniques were not necessary. Importantly, no ischemic complications were encountered.

**Conclusion:**

This case report describes the successful combination of preoperative celiac axis stenting, NAC, and surgical intervention for the management of CAS in an elderly patient with pancreatic cancer. This approach offers a potential solution for maintaining the hepatic arterial blood flow in the presence of CAS without vascular reconstruction, particularly in elderly individuals.

## Background

Celiac axis stenosis (CAS) is a relatively common finding that has been observed in up to 27.6% of patients who undergo pancreaticoduodenectomy (PD) [1–5]. For patients with CAS, the hepatic arterial flow is supplied via the gastroduodenal artery (GDA) through the pancreatic head arcade from the superior mesenteric artery, and the GDA needs to be dissected during PD. Al-Saeedi stated that severe CAS was associated with an increased risk for clinically relevant complications [6]. Therefore, for patients with CAS undergoing PD, preoperative recanalization or intraoperative vascular reconstruction of the celiac axis is necessary to maintain the hepatic arterial blood flow. We describe a patient who underwent PD after preoperative endovascular celiac axis stenting and neoadjuvant chemotherapy (NAC).

## Case presentation

**Fig. 1:**
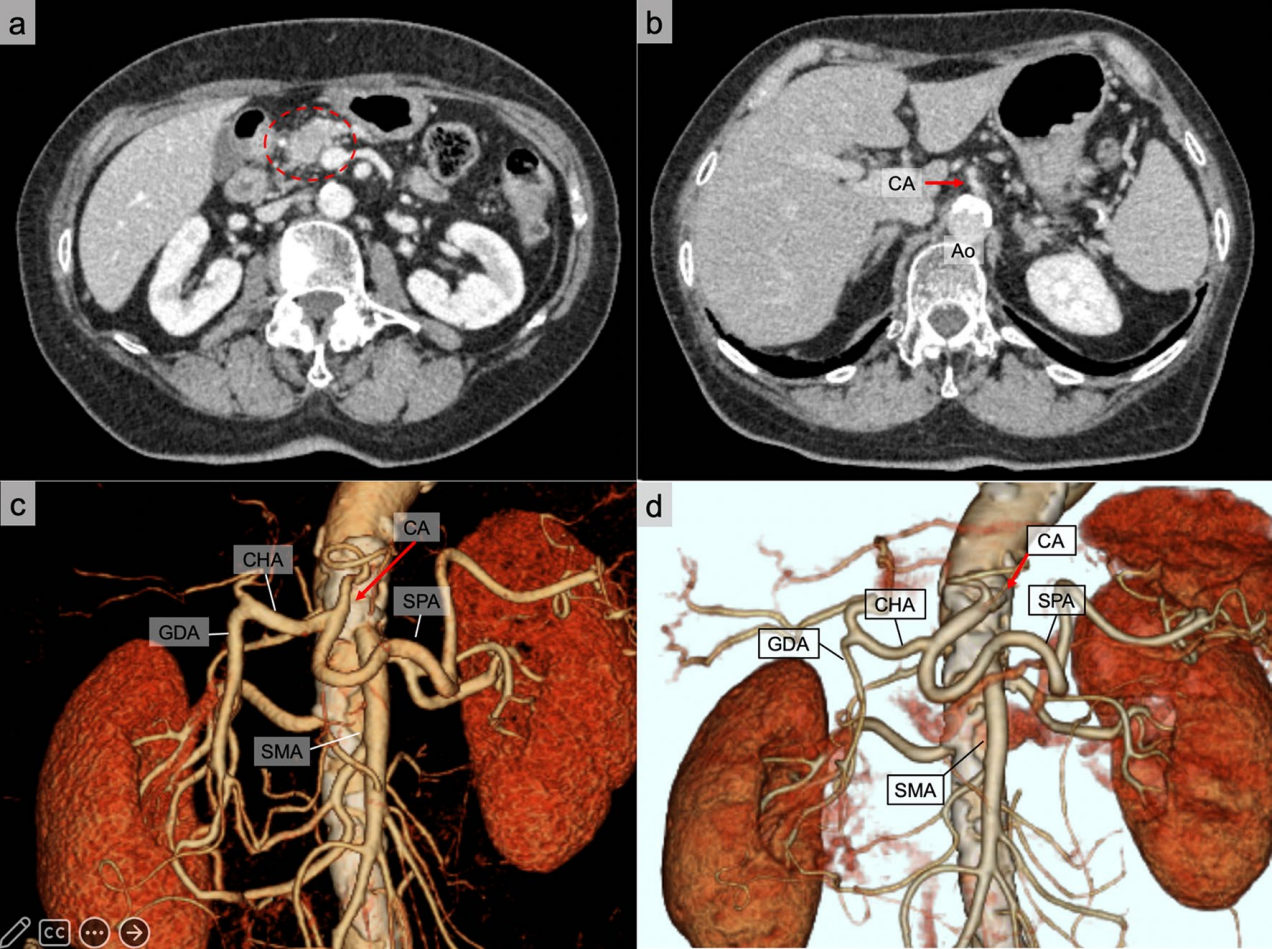
Axial view and three-dimensional reconstruction using contrast-enhanced computed tomography (CT). **a** An 11 mm × 15 mm low-density mass in the posterior aspect of the pancreatic body with attachment to the superior mesenteric vein (SMV) of less than 180 degrees. **b** Celiac axis stenosis (CAS) caused by severe calcification. **c** Well-developed collateral flow from the superior mesenteric artery (SMA) to the gastroduodenal artery (GDA). **d** After stent placement, the pancreatic arcade became narrower and the PHA became thicker

An 81-year-old woman with pancreatic duct dilatation was referred to our hospital. Contrast-enhanced computed tomography (CT) revealed an 11 mm × 15 mm low-density mass in the posterior aspect of the pancreatic head. Approximately less than 180 degrees of the tumor was in contact with the superior mesenteric vein (Fig. 1a). The tumor had not invaded the common hepatic artery, celiac axis, or superior mesenteric artery (SMA). No metastatic lymph nodes or organs were detected. Abdominal CT without contrast revealed multiple calcifications in the aorta and visceral arteries. Furthermore, the celiac axis showed severe stenosis caused by calcification (Fig. 1b). Although the arterial pathway between the proper hepatic artery and SMA developed through the GDA, we identified well-developed collateral flow from the SMA to the GDA via the inferior pancreatic duodenal artery (Fig. 1c). The tumor marker levels (CEA, CA19-9, DUPAN-2, Span-1) were within the normal limit. Endoscopic ultrasound-guided fine-needle aspiration revealed adenocarcinoma. The patient was diagnosed with T1, N0, M0, and stage IA pancreatic head cancer according to the 8th edition of the Union for International Cancer Control TNM classification. In previous papers, vascular reconstruction with PD increases the risk of thromboembolism and postoperative bleeding (caused by pancreatic fistula) [7–9] and, this patient had no suitable bypass vessels available because of severe arteriosclerosis. Thus, we planned to perform surgery after stent placement and NAC. Prior to PD, we consulted with an interventional cardiologist to perform percutaneous transluminal angioplasty on the celiac axis.

Angiography of the abdominal aorta did not reveal a celiac axis. An antegrade approach directly from the aorta to the celiac axis is difficult because of severe stenosis or occlusion. Hence, our strategy involved retrograde access to the celiac axis through a collateral pathway originating from the SMA.

**Fig. 2:**
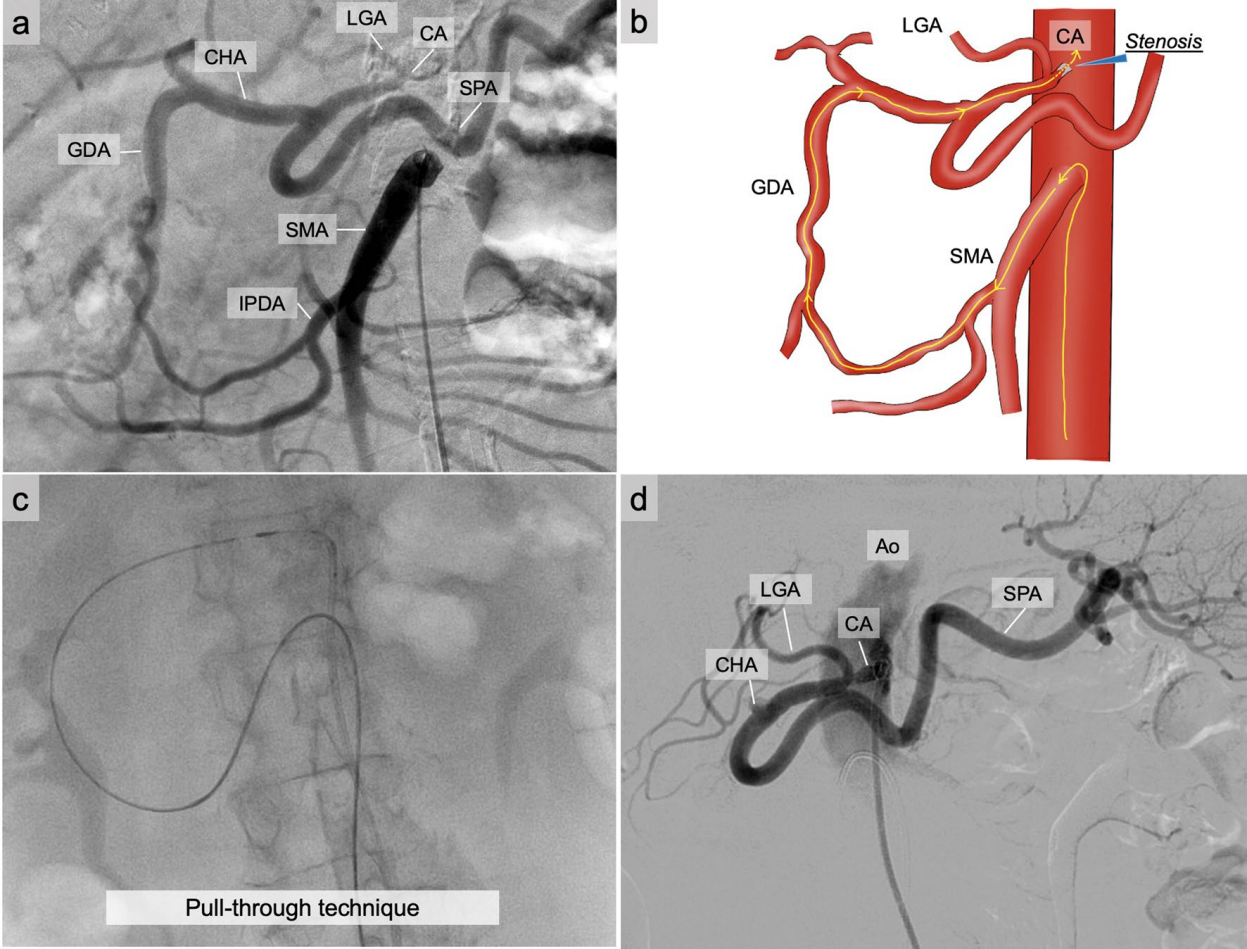
Preoperative angiography. **a** The celiac axis (CA) was retrogradely visualized through the collateral pathway originating from the superior mesenteric artery (SMA). **b**, **c** The microguidewire was antegradely inserted in the CA from the SMA using the pull-through technique. **d** Angiography of the CA revealed the hepatic artery and splenic artery

Percutaneous revascularization of the celiac axis was performed using a right femoral artery approach. A 7-Fr guiding catheter was selectively inserted in the SMA. The celiac axis was retrogradely visualized through the collateral pathway originating from the SMA (Fig. 2a). Subsequently, a triple coaxial system was used to reach the celiac axis. Severe CAS posed a significant challenge in advancing the micro-guidewire; however, through diligent effort, we were able to successfully navigate the wire through the celiac axis. A 6-Fr guide catheter was positioned at the entrance of the abdominal aorta, and the microguidewire was grasped from the aorta using a snare. Then, a microguidewire was inserted antegradely in the celiac axis from the left femoral artery using the pull-through technique (Fig. 2b, c). A balloon catheter was used to dilate the celiac axis. Careful handling was necessary in situations with a high risk of lacerations, because calcification might cause the arterial walls to harden, reducing their flexibility. When intravascular ultrasound was used to visualize the celiac axis, severe calcification was observed throughout its circumference. A balloon-expandable stent was successfully deployed in the celiac axis, and stent dilatation was performed using a balloon dilatation catheter. Contrast imaging of the celiac axis revealed the hepatic and splenic arteries (Fig. 2d); however, the GDA was not visualized because of the accordion phenomenon. GDA ligation did not seem to impede the hepatic blood flow from the celiac axis. The stent was placed so as not to cross the left gastric artery (LGA). After stent placement, the arcade of pancreas head became narrower and the proper hepatic artery (PHA) became thicker (Fig. 1d). This suggested increased blood flow from the CA.

**Fig. 3:**
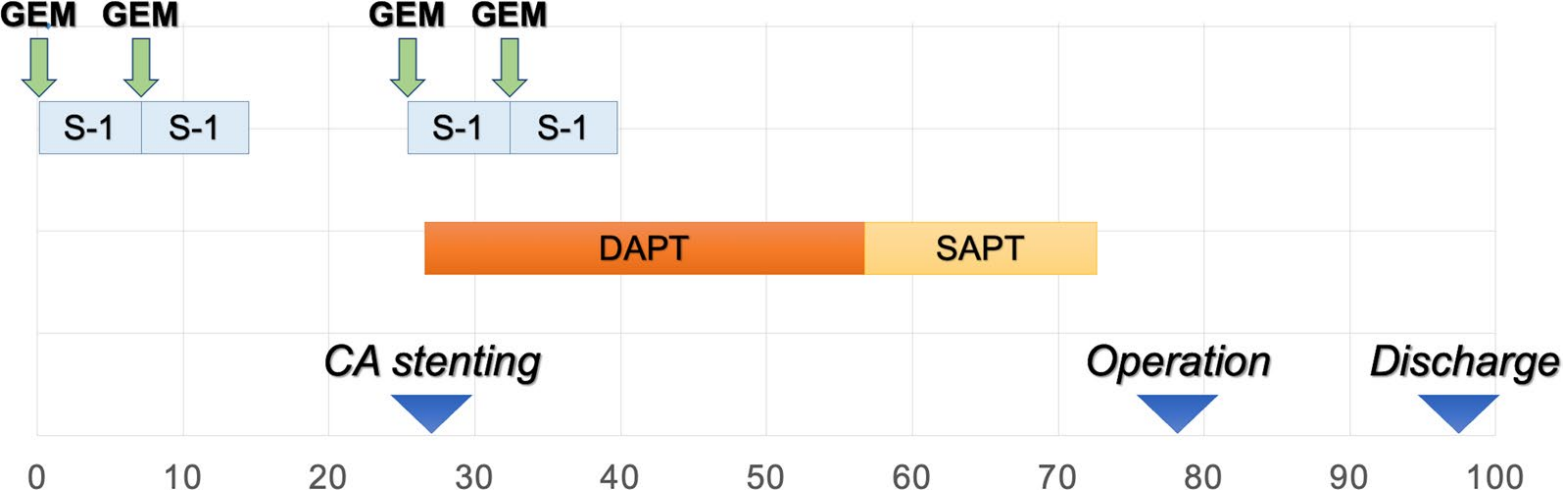
Preoperative progress

The patient received two cycles of neoadjuvant Gemcitabine (GEM) and S-1. One month after starting chemotherapy, we placed a stent and started 1 month of dual antiplatelet therapy (DAPT) (100 mg oral aspirin and 30 mg direct factor Xa inhibitors). Subsequently, DAPT was switched to single antiplatelet therapy (30 mg direct factor Xa inhibitors). The preoperative progress is presented in Fig. 3.

**Fig. 4:**
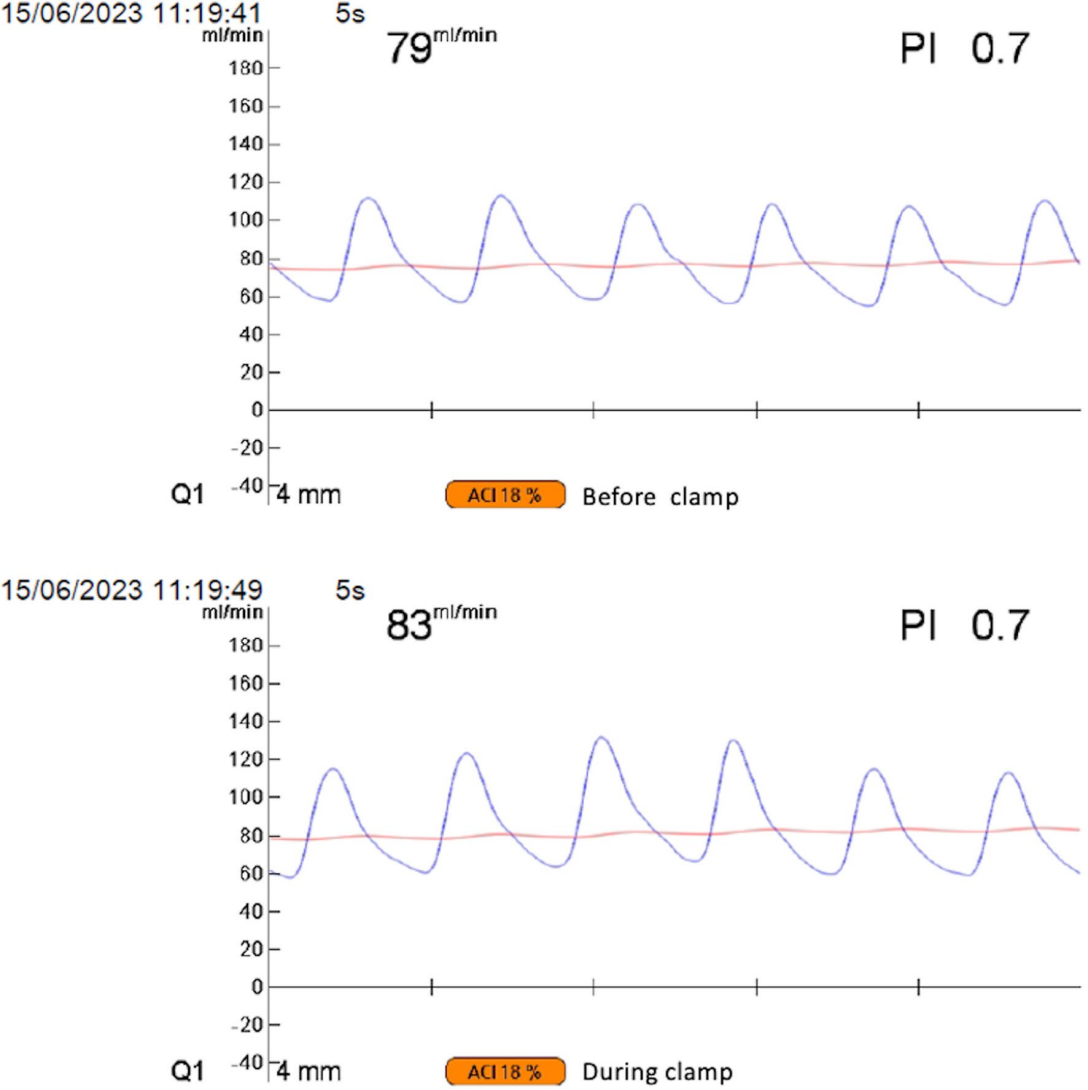
Monitoring of the arterial blood flow

After two cycles of NAC and stent placement, subtotal stomach-preserving PD was performed. After clamping the GDA, we intraoperatively confirmed that the hepatic artery blood flow was adequately maintained by monitoring the flow velocity and direction of blood flow using a blood flow meter (Fig. 4); then, we dissected the GDA. The surgical procedure was routinely performed without using any special techniques. Grade A chylous ascites developed postoperatively, and the patient was discharged on day 17.

## Discussion

The causes of CAS can be divided into three categories: (1) extrinsic compression by median accurate ligament (MAL), celiac ganglion, or surrounding fibroinflammatory tissue; (2) intrinsic stenosis caused by arteriosclerosis; (3) other causes such as congenital factors, acute and chronic dissection, neoplastic invasion, compression by an acutely inflamed pancreas (in cases of pancreatitis) or a large tumor, and injury during angiography. The most common cause of CAS in Western countries is arteriosclerosis and that in Eastern countries is MAL compression [10, 11]. Based on the CT results, the cause of severe CAS in this case was strongly suspected to be arteriosclerosis. This elderly patient experienced a favorable outcome as a result of preoperative stent placement and NAC.

Mitigation strategies: preoperative radiologic intervention or chemotherapy, MAL division in cases of extrinsic CAS, and even vascular reconstruction, play a crucial role, because higher grade of CAS is associated with an increased risk for clinically relevant complications such as POPF, liver perfusion failure and gastric ischemia [6]. For patients with MAL compression undergoing PD, division of the MAL during surgery can be considered as the primary procedure. CAS caused by arteriosclerosis is treated with bypass grafting, endarterectomy, or preoperative stenting. Elderly patients with severe stenosis or complete occlusion are not eligible for surgical reconstruction because vascular reconstruction with PD increased 30-day postoperative morbidity and mortality [12]. When CAS is suspected to be caused by atherosclerosis in elderly individuals, preoperative stent placement without MAL division is a favorable choice.

Combined therapy comprising stent placement and NAC is beneficial for elderly patients with CAS caused by arteriosclerosis. The timing of initiating NAC and stent placement is crucial. NAC has gained widespread acceptance as a standard treatment for pancreatic cancer, and it has received endorsement from the National Comprehensive Cancer Network guidelines [13]. Typically, NAC is administered at 2- to 3-month intervals before surgical intervention. Additionally, after stent placement for arteriosclerosis, the JCS/JSVS 2022 Guideline on the Management of Peripheral Arterial Disease recommends DAPT for 2 to 3 months, followed by single antiplatelet therapy, to reduce the risk of blood clot formation around the stent [15, 16]. The blood flow velocity in the celiac axis is faster than that in the peripheral arteries, and it may not require several months of DAPT. For our case, we collaborated with the cardiology department to place the stent in the CAS portion while the patient was undergoing chemotherapy. After stent placement, and after 1 month of DAPT, the treatment was reduced to single antiplatelet therapy for 1 month before surgery (Fig. 3). The present case of pancreatic cancer with CAS demonstrated a favorable outcome because stent placement during preoperative chemotherapy and DAPT for 1 month enabled the safe execution of PD.

Combined therapy comprising stent placement and NAC for CAS caused by MAL has been previously reported [14]. For this case, a similar procedure was followed: stent placement was performed during chemotherapy, which was followed by surgery. Twelve weeks of DAPT was administered after stent placement, followed by heparin bridging therapy and PD. The timing of stent placement, duration of DAPT, and appropriate timing of surgery remain unknown. Reports of combination therapy involving chemotherapy and stent placement are limited to only two cases, including ours. Therefore, there is ongoing debate regarding the optimal timing of stent placement.

## Conclusions

An approach comprising chemotherapy and stent placement followed by surgical intervention for elderly patients with pancreatic cancer and CAS has been proven to be beneficial.

## Data Availability

The datasets supporting the conclusions of this case report are included within the article and its supplementary materials. Any additional information or raw data are available from the corresponding author upon reasonable request.

## Abbreviations

CAS: Celiac axis stenosis
CT: Computed tomography
DAPT: Dual antiplatelet therapy
SAPT: Single antiplatelet therapy
GDA: Gastroduodenal artery
MAL: Median accurate ligament
NAC: Neoadjuvant chemotherapy
GEM: Gemcitabine
PD: Pancreaticoduodenectomy
SMA: Superior mesenteric artery
PHA: Proper hepatic artery
SPA: Splenic artery
CHA: Common hepatic artery
IPDA: Inferior pancreaticoduodenal artery
PHA: Proper hepatic artery
LGA: Left gastric artery
